# Refractory Dysphagia From Stent-Induced Esophageal Ring Formation After Sequential Bariatric Surgery: A Case Report

**DOI:** 10.7759/cureus.107229

**Published:** 2026-04-17

**Authors:** Iten I Tanyous, Ann I Tanyous, Alaa-Salah Eldin

**Affiliations:** 1 Department of Gastroenterology, Division of Internal Medicine, Touro College of Osteopathic Medicine, Middletown, USA; 2 Department of Gastroenterology, Division of Internal Medicine, Hudson Regional Hospital, Secaucus, USA

**Keywords:** balloon dilation, esophageal stricture, esophagitis, gastric bypass complications, malnutrition, postbariatric surgery, stent-related complications

## Abstract

A 55-year-old female patient with prior sleeve gastrectomy and subsequent Roux-en-Y gastric bypass conversion presented with progressive dysphagia. Several years prior, a self-expanding metal stent had been placed to manage postbariatric dysphagia. The patient subsequently developed a proximal esophageal B-ring with critical luminal narrowing at the superior margin of the stent. This complication is described in the literature but may be underrecognized in postbariatric patients. Two esophageal balloon dilations provided only temporary relief before stricture recurrence. The clinical course was complicated by upper gastrointestinal bleeding, progressive anemia, and severe malnutrition requiring prolonged total parenteral nutrition. Given the failure of nonoperative interventions, the patient underwent exploratory laparotomy with surgical jejunostomy placement. At the two-week follow-up, the patient tolerated tube feeds and demonstrated early nutritional recovery. The mechanism is hypothesized to involve fibrosis from chronic mechanical irritation at the stent margin, potentially accelerated in postbariatric patients due to nutritional deficiencies and altered esophageal physiology. Clinicians should be aware that new stricture formation has been reported following benign esophageal stent placement. Close endoscopic surveillance of the proximal stent margin may permit early detection of reactive tissue changes in this population.

## Introduction

Esophageal stenting is an established intervention for both malignant and benign esophageal conditions, though its use for benign disease remains controversial due to complications such as migration, tissue ingrowth, and new stricture formation at stent margins [1]. Postbariatric patients with altered gastrointestinal anatomy represent a particularly challenging population, as the combination of complex surgical reconstruction and chronic nutritional deficiencies creates a hostile environment for stent placement and limits therapeutic options when complications arise [2]. New stricture formation proximal to esophageal stents has been reported, though its frequency in patients with reconstructed bariatric anatomy is not well-characterized [1,3]. We present the case of a 55-year-old female patient with prior sleeve gastrectomy and Roux-en-Y gastric bypass conversion who developed a symptomatic proximal esophageal B-ring following esophageal stent placement, ultimately requiring surgical intervention after failure of multiple endoscopic therapies. This case highlights the unique challenges of managing stent-related strictures in postbariatric patients and emphasizes the need for careful surveillance following esophageal stent placement in this population.

## Case presentation

​​A 55-year-old female patient presented with progressive dysphagia and complete inability to tolerate oral intake over a 14-month period. Her surgical history included a sleeve gastrectomy performed eight years prior, with subsequent conversion to Roux-en-Y gastric bypass five years prior. She had also undergone open splenectomy and cholecystectomy. Home medications included pantoprazole, sucralfate, levothyroxine, duloxetine, and clonazepam. She reported allergies to penicillin and shellfish.

Three years prior to presentation, the patient had undergone esophageal stent placement to address postbariatric dysphagia. The stent was associated with the development of a proximal esophageal ring causing critical luminal narrowing, representing a rare but recognized complication. At our institution, she underwent two esophageal balloon dilations six months apart, each providing only transient symptomatic improvement before stricture recurrence.

On physical examination, the patient appeared cachectic with marked muscle wasting, reflecting prolonged inadequate nutritional intake. Vital signs were stable. Mucous membranes were pale. The abdomen was soft with hypoactive bowel sounds, without tenderness, masses, or organomegaly.

Laboratory evaluation demonstrated significant anemia with a hemoglobin count of 9.1 g/dL and a hematocrit count of 27.4%, consistent with chronic blood loss and malnutrition. White blood cell and platelet counts were within normal limits. Computed tomography of the abdomen and pelvis with intravenous contrast, performed on admission, revealed marked circumferential wall thickening of the distal esophagus and gastroesophageal junction, findings that had progressed compared to prior imaging obtained six months earlier. The differential diagnosis included infectious, inflammatory, or neoplastic etiologies. Additional findings included high-grade stenosis of the celiac artery origin, small bilateral pleural effusions with left lower lobe collapse, and postsurgical anatomical changes consistent with her bariatric history.

**Figure 1:**
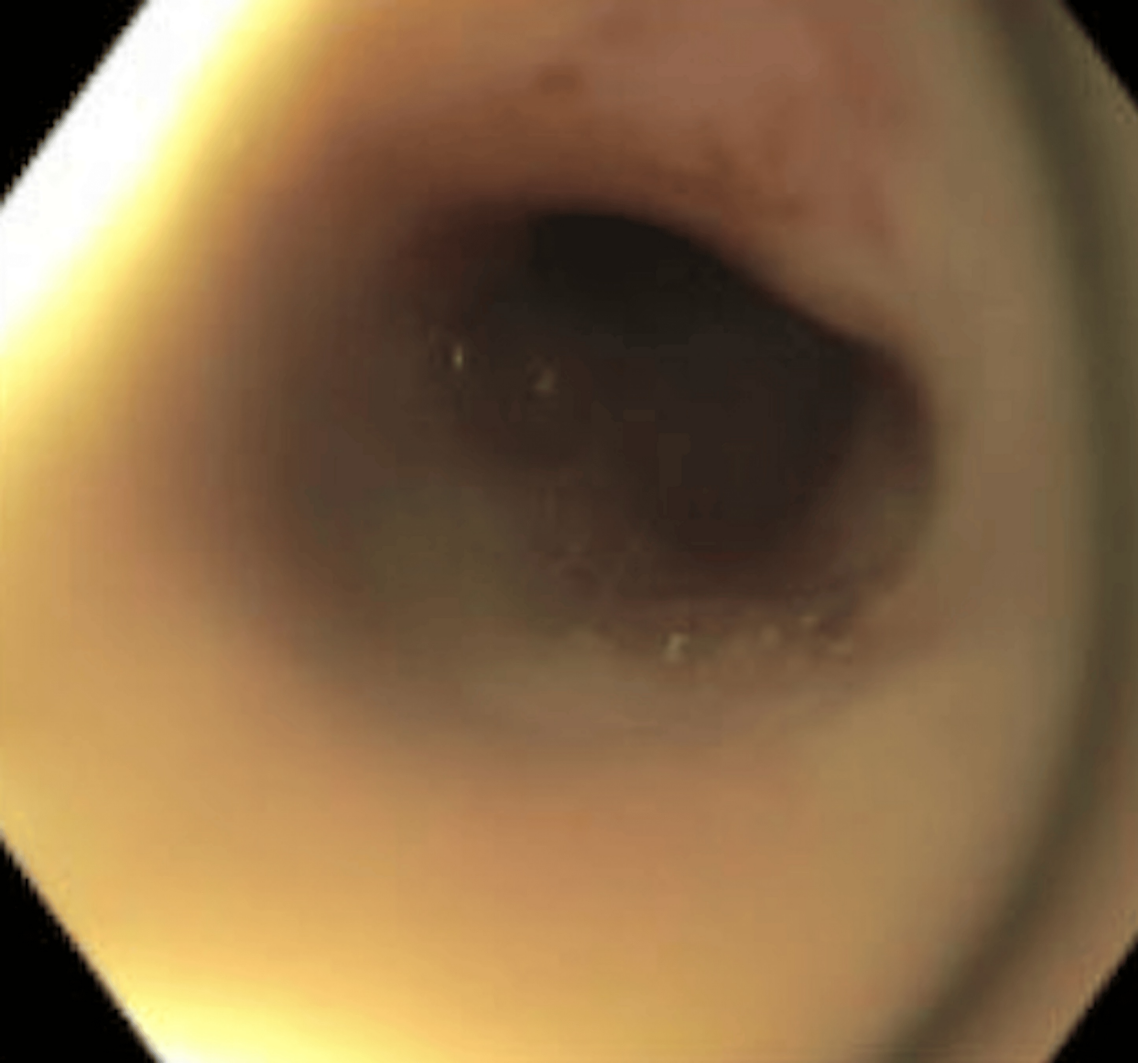
Esophagogastroduodenoscopy demonstrating a proximal esophageal B-ring with significant luminal narrowing

**Figure 2:**
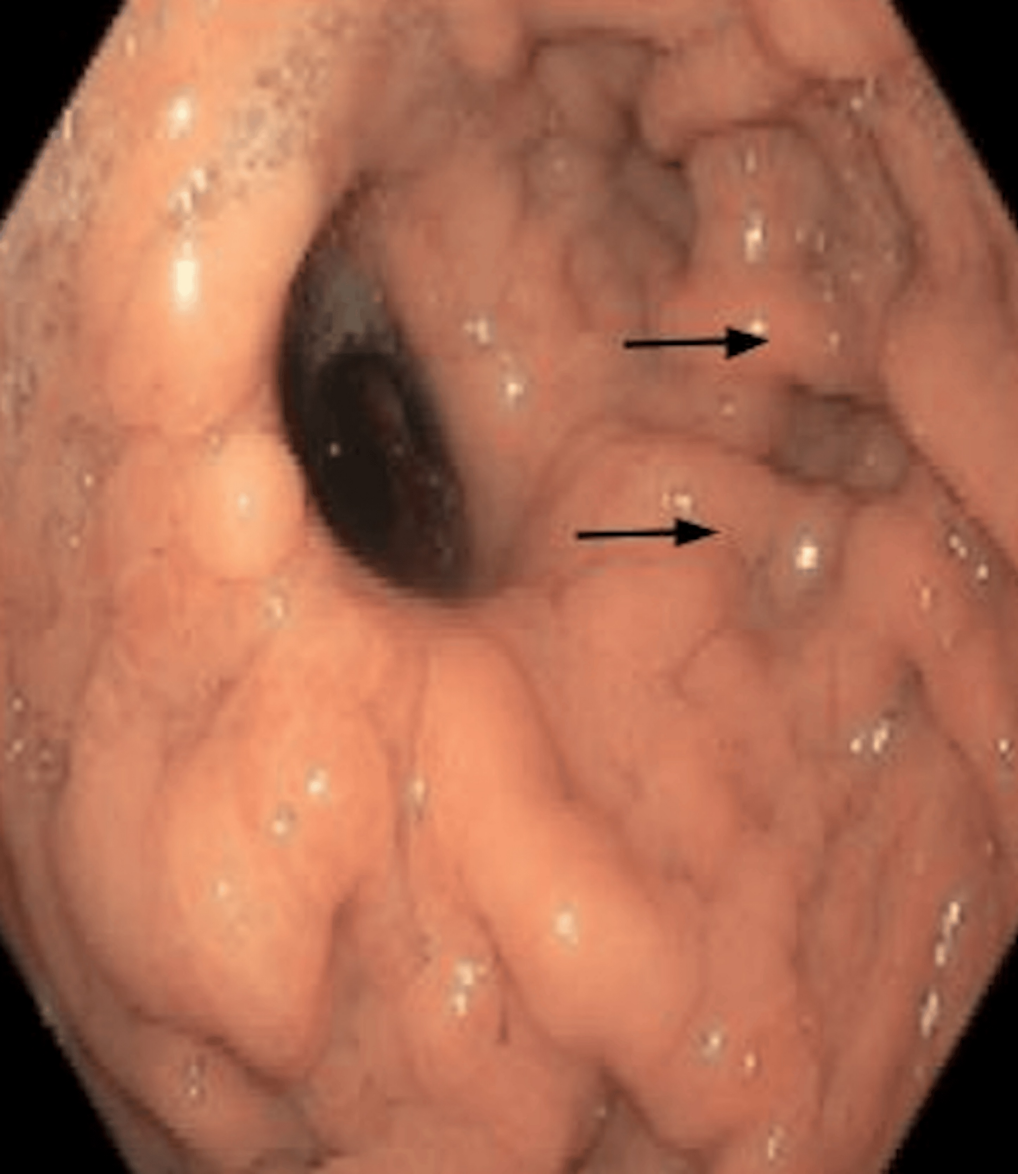
Endoscopic view of the stomach showing a small area of mucosal hyperplasia as indicated by the black arrows

Esophagogastroduodenoscopy confirmed a proximal esophageal B-ring with significant luminal narrowing (Figure 1). Endoscopic biopsies demonstrated benign inflammatory changes without evidence of malignancy or infection. Examination of the stomach revealed a small area of mucosal hyperplasia (Figure 2, black arrows). Despite repeat balloon dilations, the stricture recurred, demonstrating refractoriness to standard endoscopic therapy. Interventional radiology attempted nasoenteric tube placement for enteral access; however, this was unsuccessful due to the inability to safely advance a guidewire through the obstructed and surgically altered anatomy.

The patient's clinical course was further complicated by her complete dependence on total parenteral nutrition for several months, placing her at risk for catheter-related infections and metabolic complications. A multidisciplinary conference involving gastroenterology, bariatric surgery, interventional radiology, and clinical nutrition determined that surgical intervention was the only remaining option. The patient and her daughter were counseled extensively regarding the risks associated with operative intervention in the setting of severe malnutrition and complex adhesive disease. After a thorough discussion, informed consent was obtained.

**Figure 3:**
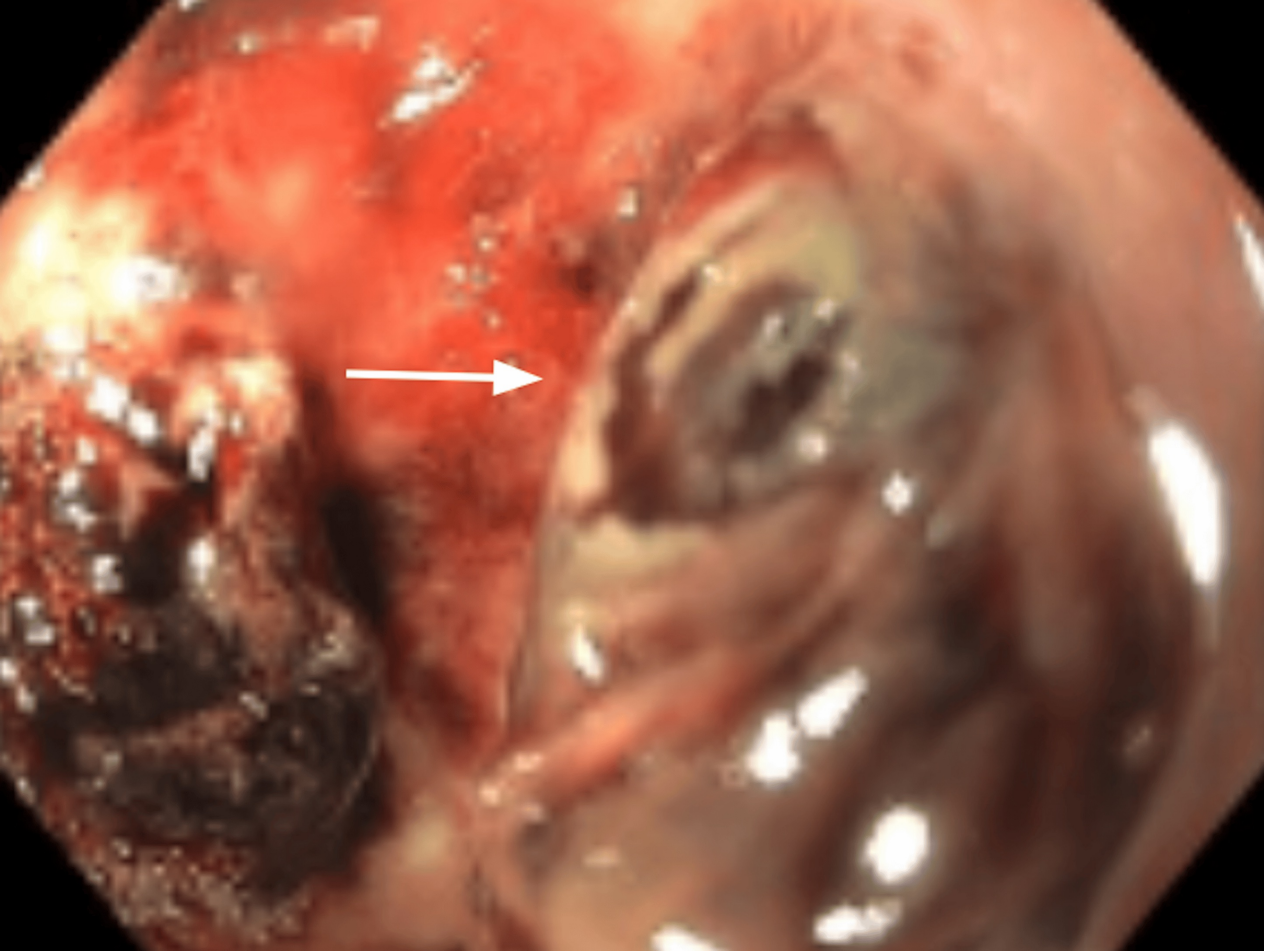
Endoscopic examination at the jejunal feeding tube insertion site revealing chronic thrombosis and inflammation surrounding the surgical site White arrow indicates the mushroom head of the feeding tube centrally embedded within the thrombotic tissue

The patient underwent exploratory laparotomy with extensive lysis of adhesions and placement of a surgical feeding jejunostomy tube. Endoscopic examination at the jejunal insertion site revealed chronic thrombosis and inflammation surrounding the tube, with the mushroom head centrally embedded within thrombotic tissue (Figure 3). Postoperative computed tomography with oral contrast confirmed appropriate tube positioning without evidence of leak or bowel perforation.

At the two-week follow-up, the patient demonstrated significant clinical improvement. She was tolerating jejunostomy tube feedings well without evidence of gastrointestinal bleeding or anterior abdominal wall cellulitis. Peripheral hyperalimentation was continued to supplement enteral nutrition during the transition period. Physical examination revealed stable vital signs, a well-functioning feeding tube, and no signs of peritonitis. The patient reported resolution of her chronic back pain and was actively participating in physical therapy for nutritional rehabilitation and functional reconditioning.

## Discussion

This case highlights a rare iatrogenic complication: proximal esophageal B-ring formation induced by an indwelling esophageal stent in a post-bariatric surgery patient. This represents a rare presentation not well-described in patients with prior sleeve gastrectomy and Roux-en-Y gastric bypass, adding to the limited literature on long-term esophageal sequelae following bariatric surgery.

The mechanism of stent-related benign esophageal injury is well-described. Fibrosis secondary to chronic mechanical irritation and granulation tissue formation at stent margins are recognized complications of esophageal stenting, particularly in benign disease [1]. These processes may lead to luminal narrowing and reactive stricture formation adjacent to the stent edge. In this patient, the temporal association between stent placement and development of a proximal B-ring suggests chronic mechanical injury as a likely contributor to pathology [1].

Patients with prior bariatric surgery represent a high-risk population for esophageal dysfunction. Postoperative dysphagia and esophageal motility disorders have been reported following both sleeve gastrectomy and Roux-en-Y gastric bypass [2]. Sleeve gastrectomy in particular has been associated with increased esophageal acid exposure and a higher incidence of erosive esophagitis, which may further predispose to mucosal injury and fibrosis [2]. In this context, altered esophageal physiology combined with chronic mechanical irritation from an indwelling stent may have contributed synergistically to stricture formation.

Although ringlike esophageal strictures are most commonly located distally, similar fibrotic remodeling may occur at other sites of chronic irritation, particularly in the presence of foreign bodies such as esophageal stents and in patients with complex foregut anatomy [1,3].

Our patient met the criteria for refractory benign esophageal stricture, defined as the inability to maintain a luminal diameter of 14 mm for at least four weeks following adequate dilation [3]. Endoscopic management of refractory strictures remains challenging. Temporary stent placement has reported clinical success in approximately half of patients; however, recurrence and new stricture formation remain common complications [4]. In this case, repeated balloon dilations provided only transient relief, consistent with the limited durability of endoscopic therapy in complex benign strictures [4].

This case also underscores the potential for significant downstream complications, including malnutrition and anemia, when refractory esophageal obstruction persists. Surgical jejunostomy provided definitive enteral access after failure of endoscopic therapy, highlighting the importance of early multidisciplinary involvement in complex cases.

Several limitations should be acknowledged. Details regarding the original stent type, duration of placement, and indication were not available. Histopathological confirmation of fibrosis was not obtained, which limits definitive mechanistic conclusions.

## Conclusions

This case illustrates an important complication: an esophageal stent deployed to alleviate post-bariatric dysphagia instead induced proximal B-ring formation, ultimately necessitating surgical intervention. The cascade of complications, including refractory stricture, progressive malnutrition, and failed endoscopic therapies, underscores the trajectory that can unfold when standard interventions fail in patients with surgically altered anatomy. Three lessons emerge for clinicians: esophageal stent placement in postbariatric patients warrants close endoscopic surveillance of stent margins; early multidisciplinary consultation should not be delayed when nonoperative measures fail; and prospective studies are needed to guide evidence-based management in this growing population.
